# Miscellaneous

**Published:** 1873-11

**Authors:** 


### Miscellaneous.

### THE BIG TELESCOPE.

We always feel repaid for a visit of half an hour to the workshop of our friend, Alvan Clark ; for he is a man thoroughly in love with his work, and ennobles the work while he is ennobled by it.

The large telescope for the Naval Observatory is rapidly approaching completion, and it is hoped that it will be in place by the end of September. The tube is now being manufactured. This is of sheet steel, and built up in such a way that its largest diameter is at the point of suspension, each succeeding segment towards either end fitting into the one preceding it; so that the tube will, when completed, be cigar-shaped. Work on the large instrument has been delayed somewhat, from the fact that work on the small transit instruments for observing the transit of Venus admits of no delay. The Messrs. Clark are constructing ten of these instruments ; eight of them w l be accompanied by chronographs, which they will also build. These telescopes are all adjustable for polar distance; since, being intended for use in various latitudes, they cannot be adjusted permanently, as in the case when the exact position of the observatory is known. They are run by clock movements, which are reversible, thus fitting them for north or south latitude.

Mr. Clark is also at work on the glass stolen about a year ago from the Alleghany Observatory, correcting its figure and repolishing it, as it was somewhat damaged while in possession of the thieves, who buried it in the ground. He has just sold the twelve-inch glass which he has had on hand for a year or two past. This is to go, we believe, to the Imperial Observatory at Vienna. The glass for the second large telescope is not yet made, but they are expecting word that it is ready for their inspection. S.

### PHENOMENA OF THE BRAIN.

One of the most inconceivable things in the nature of the brain is that although the organ of sensation, it should itself be insensible. To cut the brain gives no pain; yet in the brain resides the power of feeling pain in any part of the body. If the nerve which leads to it from the injured part be divided, we become instantly unconscious of suffering. It is only by communication with the brain that any kind of sensation is produced; yet the organ is itself insensible. But there is a circumstance more wonderful still. A certain portion of the brain itself may be removed without destroying life. The animal lives and performs all those functions which are necessary to simple vitality, but it has no longer a mind. It can not think or feel. It requires that the food should be pushed into its stomach; once there, it is digested, and the animal will even thrive and grow fat. We infer, therefore, that a part of the brain is simply intended for the exercise of the intellectual faculties, whether of the lower degree, called instinct, or of that exalted kind bestowed on man, called reason.—X

### THE ADMINISTRATION OF PERCHLORIDE OF IRON.

Dr. H. L. Snow remarks that delicate patients very frequently object to the astringent metallic taste long remaining in the mouth after the administration of tincture of perchloride of iron, the flavor of which is but very imperfectly disguised by the syrup or spiritus chloroformi with which it is usually ordered. It may not, perhaps, be too insignificant a matter for mention, that the substitution of a small quantity of glycerine (about half an ounce to an eight-ounce mixture) will altogether obviate this inconvenience.—*Med. & Sur. Rep.*

